# Review of Emotion Regulation in Late Life Mood Disorders

**DOI:** 10.20900/jpbs.20240008

**Published:** 2024-11-22

**Authors:** Regan E. Patrick, Rebecca A. Dickinson, Allison Gregg, Jack R. Kaufman, Jeremy Maciarz, Julia G. Merrill, Loreal A. Williams, Sara L. Weisenbach

**Affiliations:** 1 Department of Neuropsychology, McLean Hospital, Belmont, MA 02478, USA; 2 Division of Geriatric Psychiatry, McLean Hospital, Belmont, MA 02478, USA; 3 Department of Psychiatry, Harvard Medical School, Boston, MA 02215, USA; 4 Department of Psychiatry, University of Texas Southwestern Medical Center, Dallas, TX 75390, USA

**Keywords:** emotion regulation, late life depression, older adult bipolar disorder, executive function

## Abstract

Emotion regulation (ER), or the ability to modulate the experience and expression of emotion, is critical to adaptive functioning and is a key feature of mood disorders. At the same time, normal aging is associated with changes in ER, though the interaction of aging with the presence of a mood disorder are unclear. Here, we review what is known about ER and its underlying neural mechanisms in late life mood disorders, specifically late life depression and bipolar disorder. We also review behavioral and neuromodulation therapies that seek to reduce negative affect and improve positive affect. We conclude with recommendations for future research into the nature and mechanisms of ER and interventions targeting ER in older adults with mood disorders.

## SEARCH STRATEGY

The current Mini Review considered research articles, meta-analyses, and reviews published in English and indexed on PubMed/MEDLINE and Web of Science up to April 2024. Our initial search did not include any restriction on earlier publication date; however, 80% of included citations were published within the past 10 years (since 2014). Specific search terms included “emotion regulation” in combination with other terms specific to the content of each section, including (but not limited to): “late life depression”, “older adult bipolar disorder”, “normal aging”, “executive function”, “cognitive control network”, “dorsal anterior cingulate cortex”, “psychotherapy”, “neuromodulation”, “neurotransmitter”, “transcranial magnetic stimulation”, and “social engagement”. We also reviewed reference lists of select articles identified in our search (e.g., meta-analyses and systemic reviews) and included those that were deemed relevant.

## EMOTION REGULATION: DEFINITION & NEUROCOGNITIVE MECHANISM

**Figure 1.**
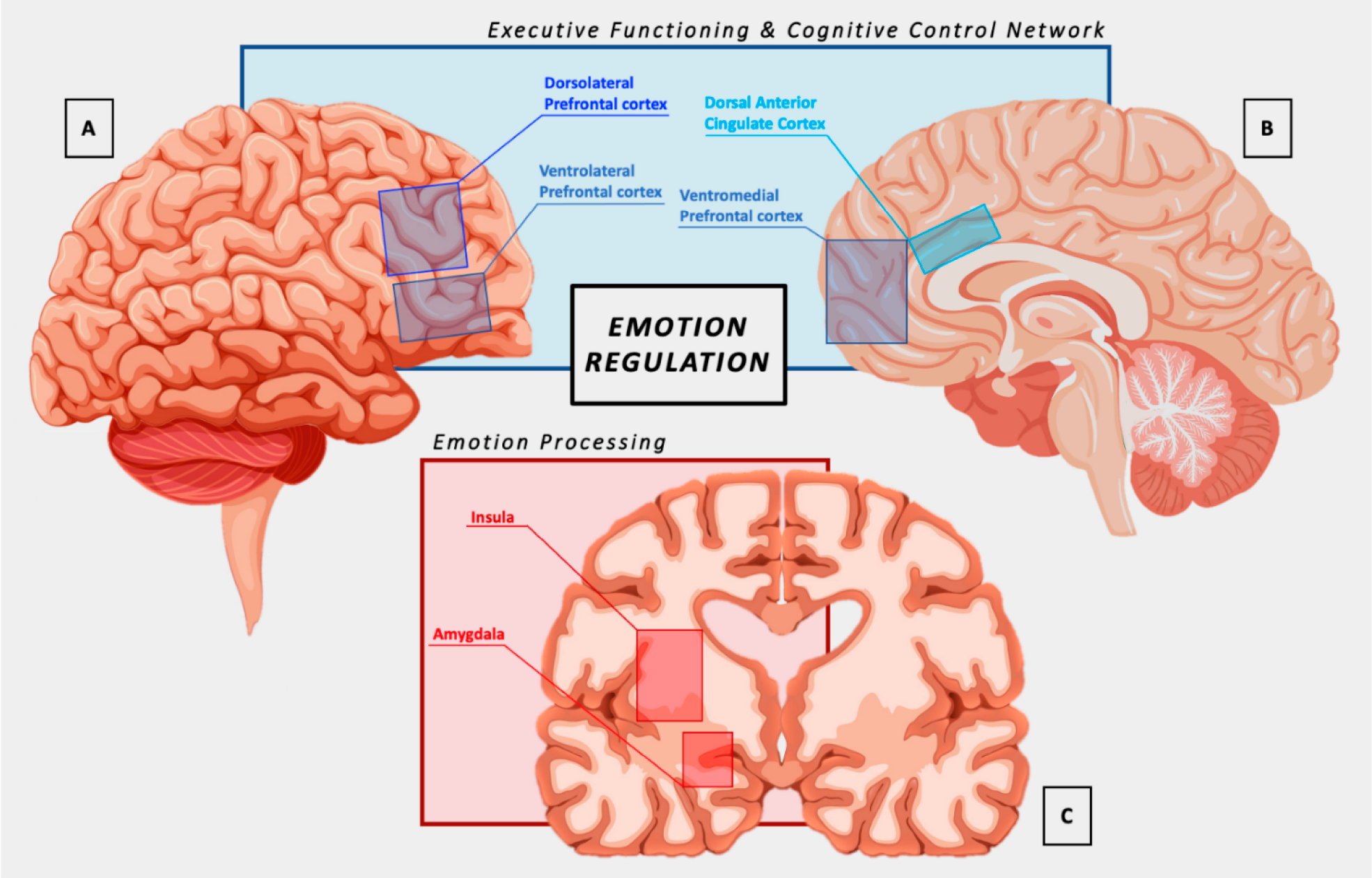
Brain regions involved in emotion regulation: (**A**) sagittal lateral view; (**B**) sagittal medial view; and (**C**) coronal view. Regions shown in (A) and (B) are primarily implicated in the top-down regulation of regions in (C).

Emotion regulation (ER) refers to processes that enable us to modulate the experience and expression of emotion, which is critical for adaptive functioning [1]. Many ER strategies have been identified, ranging from simple distraction away from emotionally charged information to higher-level cognitive reappraisal—i.e., reinterpreting emotional information to change its meaning and consequent impact [2]. Successfully deploying ER strategies partly depends on the use of executive function (EF) skills, which comprise a range of cognitive processes that allow for the organization, planning, monitoring, and successful execution of goal-directed behaviors. Accordingly, there is considerable overlap in the neural networks that underlie both ER and EF, with the cognitive control network (CCN) being central in this regard (Figure 1). For example, the prefrontal cortex, including the dorsolateral prefrontal cortex (DLPFC) and ventrolateral prefrontal cortex (VLPFC), plays a key role in the cognitive control of emotions, such as through cognitive reappraisal strategies [3,4]. The amygdala is another critical structure, with its activity being modulated during the regulation of emotions [5]. The dorsal anterior cingulate cortex (dACC) is specifically implicated in the integration of emotional and cognitive processes and may play a central role in ER [3]. The insula, particularly the anterior portion, is also involved in awareness and regulation of emotional states, as well as linking emotional outcomes to motivational states [3]. Functional connectivity studies have demonstrated that ER involves dynamic interactions across this network of cortical and subcortical/limbic regions [6].

## EMOTION REGULATION IN NORMAL AGING

Normal aging is associated with changes in ER across the lifespan [7]. As individuals progress through later stages of life, they can encounter myriad challenges that have a detrimental impact on emotional well-being, such as accumulating medical comorbidity burden, the decline of cognitive abilities, and the loss of loved ones [8]. Despite potential declines in physical and cognitive health, however, older adults tend to experience greater emotional stability compared to younger cohorts [9,10]. Indeed, research indicates that older adults are better able to modulate the outward expression and internal control of anger using calming strategies [11]. Similarly, older adults tend to be less reactive to negative situations, are more likely to ignore irrelevant negative stimuli, and have stronger memory for positive versus negative information [12].

These and other related findings illustrate the age-related positivity effect, wherein normal aging is characterized by a shift towards more positive affect [12], which may partly relate to differential use of ER strategies compared to young adults. For example, some studies have found that older adults use a wider range of ER strategies in response to negative affect states [13], such as greater self-reported use of acceptance in situations that evoke feelings of anxiety and sadness, whereas cognitive strategies (e.g., reappraisal) did not differ between older and younger adults [14]. More recent research has replicated the finding of greater use of acceptance in an older adult sample; however, they also exhibited less ER flexibility—a combination of ER variability (i.e., the breadth of one’s repertoire of available ER strategies) and ER context sensitivity—in their responsiveness and implementation of ER strategy use to shifts in negative affect intensity [15]. While such reduced flexibility could signal less ER variability overall, older adults may also be more selective in applying a narrower range of strategies that they know will be effective [16], including greater use of immersive-engagement strategies (e.g., perspective taking) and less use of disengagement strategies (e.g., distraction) [17].

Age-related changes in brain structure and function likely contribute to changes in ER among older adults, though the dynamics of this relationship remain an active area of investigation. Neuroimaging studies have observed that, during ER tasks involving cognitive reappraisal, increasing age predicted less negative affect and amygdala activation, as well as greater inverse coupling between ventromedial PFC (VMPFC) and amygdala [18], the latter implicating preserved top-down, frontolimbic control of emotional experience/expression. However, while older adults appear to engage similar brain structures for ER as younger adults, such as the PFC and amygdala, the efficacy of this network may be moderated by underlying cognitive/executive ability [19]. Indeed, a recent longitudinal study observed that steeper declines in EF with age were associated with both increased PFC and amygdala activity when reappraising negative stimuli, as well as diminished white matter tract integrity between these regions [20].

## EMOTION REGULATION IN LATE LIFE MOOD DISORDERS

### Late Life Depression (LLD)

Extensive research has demonstrated that major depressive disorder (MDD) is associated with differences in ER strategy use relative to healthy controls. This includes both greater use of maladaptive strategies (e.g., avoidance, rumination, and suppression) and less frequent use of more adaptive strategies (e.g., reappraisal, acceptance, and problem solving) [21]. These dynamics are at least partly related to focal difficulties with cognitive/executive control, particularly when regulating responses to negative information, which in turn can give rise to the characteristic negativity bias in MDD [22]. Moreover, neuroimaging studies suggest that individuals with MDD engage ER network regions differently than healthy controls, with reduced modulation of amygdala activation by DLPFC and VLPFC during cognitive reappraisal of negative material, as well as a disconnect between regions related to reward processing and subjective positive affect (e.g., VLPFC, ventral striatum) [23].

In LLD, age-related changes in brain structure and function that are unique to this clinical group may influence ER in maladaptive ways. A recent review of structural neuroimaging studies indicated that LLD is associated with more severe white matter disease burden and accompanying decreases in frontal-striatal-limbic network integrity, as well as decreased volume and/or cortical thickness in the PFC, orbitofrontal cortex, anterior and posterior cingulate, hippocampus, amygdala, striatum, insula, and several other cortical and subcortical regions [24]. Moreover, these imaging abnormalities were associated with cognitive dysfunction, suggesting disruption to brain networks involved in both ER and cognitive processing represent an important facet of LLD pathophysiology. Additional functional imaging data have highlighted the moderating influence of cognitive reserve (CR)—a measure of resilience against the deleterious consequences of brain pathology—on ER capacity [25]. Specifically, using education and verbal fluency as a proxy for CR, Huang et al. observed that higher CR was associated with milder depression severity, as well as stronger behavioral performance and greater medial frontal activation on an affective control task—all of which suggests that CR is related to top-down ER efficiency and thus may offer a protective buffer against age-related cognitive and emotional challenges in LLD [25]. A more recent study examined the association between premorbid personality characteristics related to emotion dysregulation, namely trait neuroticism, and structural brain changes in LLD, with smaller volume in various frontal regions being observed in those with higher neuroticism [26]. Taken together, while research directly examining ER in LLD is somewhat limited, these findings suggests that LLD is associated with altered structure and connectivity in brain networks related to ER, though various individual-level factors may have a moderating influence over how this is clinically expressed.

### Older Adult Bipolar Disorder (OABD)

As outlined above, deploying ER strategies is critical to adaptive functioning throughout the lifespan and, by extension, is an important target of investigation in affective disorders research. This is particularly relevant for bipolar spectrum disorders (BD), which are typified by emotion dysregulation and associated fluctuations in mood state, as well as reliable deficits in both EF [27] and underlying CCN activity [28,29]. Recent meta-analytic evidence indicates that BD is associated with widespread ER difficulty, including non-acceptance of emotional responses, difficulties engaging in goal-directed behavior, impulse control difficulties, and limited access to ER strategies [30]. Moreover, these ER difficulties appear to correlate with illness course, such as earlier onset and more frequent major mood episodes [31]. Interestingly, on tasks that require spontaneous ER, individuals with BD self-report greater effort, but also less perceived success, in implementing ER strategies, suggesting their heightened regulatory efforts do not translate into more affective stability [32]. Individuals with BD who are not in active major depressive or (hypo)manic states also tend to self-report more frequent rumination, catastrophizing, and self-blame, but less frequent “putting into perspective” in response to negative life events, suggesting focal difficulty in reappraising negative information [33]. Van Rheenen et al. similarly observed that poor access to mood regulation strategies, namely reappraisal, predicted depression propensity, though it should be noted that this patient sample was not uniformly euthymic (about two thirds were considered mildly depressed or hypomanic/mixed) [34]. By contrast, a recent systematic review indicated no significant behavioral differences in reappraisal between euthymic BD patients and healthy controls, though amygdala hyperactivity and aberrant frontal-amygdala connectivity during reappraisal suggested less efficient ER strategy use at a neural network level [35].

Importantly, our understanding of the cognitive and neural bases of ER in BD largely derives from studies of younger and middle-aged adults. Very little is known about ER in older adults with BD (OABD), for whom age-related changes in EF/CCN activity may interact with psychopathology and accumulated illness burden over time. While few studies have directly examined ER in OABD, other lines of evidence converge to suggest potentially unique ER dysfunction in this population. For example, the clinical trajectory of BD tends to shift toward more depressive and fewer manic episodes with age, suggesting patients are less able to regulate negative affective states specifically. In addition, although EF declines with age in both healthy individuals and OABD, the latter may be less resilient to the functional consequences given preexisting EF decrements at baseline (i.e., less EF reserve). Moreover, recent data suggest that the positivity effect in normal aging may not be present to the same degree in OABD [36], though data in this regard are sparse and variable [37]. Such inconsistent findings, in conjunction with the general dearth of research on ER in OABD, illustrate the need for further investigation.

### The Role of Neurotransmitter Systems

While an exhaustive review of the complex interplay between neurotransmitter function, emotional processing, and aging is beyond the scope of this review, a more focused overview of how these dynamics might influence ER in late life mood disorders is warranted. However, it is important to note that there is limited research to date that has directly examined the association between neurotransmitter function and ER in LLD and OABD. With that caveat in mind, LLD may be associated with several neurotransmitter changes that can impact emotional processing and ER, including the serotonergic, dopaminergic, and GABAergic systems. Serotonergic dysfunction has been observed in LLD and is characterized lower 5-HT availability across multiple cortical and subcortical regions [38]. This is noteworthy considering recent neurotransmitter mapping data that suggest brain areas implicated in executive control and cognitive reappraisal (e.g., anterior DLPFC/VLPFC and temporoparietal junction) are among the cortical regions with the highest levels of serotonin (and cannabinoid) receptors, both of which are implicated in mood regulation [39]. This suggests that serotonergic dysfunction may disproportionately contribute to ER difficulties in LLD due to the specific regional distribution of serotonin receptors and the associated functional correlates. Dopamine signaling also declines with age, and this has been linked to impairments in executive function and reward processing, both of which play important roles in the pathogenesis of LLD and can influence ER [40]. Moreover, alterations in GABAergic function within regions that are important to ER, such as the pregenual ACC, have been linked to impaired cognitive self-awareness—an important aspect of voluntary or effortful ER skills like reappraisal [41]. This suggests that GABAergic dysfunction may specifically contribute to impairments in reappraisal in LLD; however, further investigation is needed considering other data that indicates no differences in GABA-mediated cortical inhibition in older adults with and without depression [42].

Minimal research has specifically examined neurotransmitter dysfunction in OABD and the associated impact on emotion processing and regulation, though some studies may provide indirect evidence in this regard. For example, Martino et al. observed that older adults with late-onset BD exhibited higher extrapyramidal symptoms compared to early-onset BD and healthy controls [43], suggesting potentially focal dopaminergic dysfunction that could contribute to deficits in executive function and potentially ER. Other research has found increased reward-oriented impulsivity in OABD, which strongly implicates dopaminergic dysfunction affecting reward processing and decision making in the context of positive affect [44]. With respect to serotonin, a recent review of biomarkers of BD in late life highlighted the importance of genomic markers associated with serotonin metabolism, specifically implicating tryptophan hydroxylase—an enzyme involved in the synthesis of serotonin [45]. While such findings are interesting and noteworthy, the clear paucity of OABD-specific research in this area highlights the need for more focused investigation.

## TREATMENT IMPLICATIONS

### Psychotherapy

Recent research has found first session ER dynamics can serve as a prognostic tool for individuals undergoing therapy for depression [46]. At the same time, helping people with depression and other mental health conditions develop more effective ER skills is a key component is many types of psychotherapy. Such strategies include extrinsic techniques, such as watching a happy movie to increase positive affect, and intrinsic approaches, such as changing one’s perspective of an event [47]. Most psychotherapy trials, including those targeting improved ER, have been conducted in young and middle-aged populations. Given that some individuals with LLD evidence EF decline [48], it is not clear how ER skill development in younger people translates to an older population. Interventions specifically designed for older adults with MDD, such as PATH [49] and ENGAGE [50], are known to be effective in reducing depression, but do not specifically target ER or ER skill development, and outcomes on improved ER strategy use in older adult clinical samples remains understudied. With that limitation in mind, a recent study examined if/how self-reported ER strategies, namely emotional acceptance (versus detachment) and positive reappraisal, influenced the relationship between EF (i.e., working memory, inhibitory control, and verbal fluency) and depression/anxiety symptoms in healthy older adults [51]. The findings indicated that lower EF predicted higher depression and anxiety symptoms at low but not high levels of emotional acceptance, with such moderation being stronger for emotional acceptance relative to other ER strategies. While the generalizability of those results is limited because they were not derived from a clinical sample, they nonetheless highlight the potential mental health benefits of enhancing emotional acceptance in older adults who might otherwise struggle to implement more executively demanding ER strategies (e.g., reappraisal). Indeed, prior research comparing the effectiveness of reappraisal *versus* distraction in reducing negative affect in LLD found that the latter was more effective [52], perhaps because distraction has lower executive demands than reappraisal.

### Social Engagement

Social support networks are critical to maintaining a healthy lifestyle for older adults. Thus, understanding the role of ER strategy deployment in the context of social functioning can aid in the development and adaptation of effective interventions for older adults with mood disorders. Sachs-Ericsson and colleagues [53] conducted a cross-sectional study of 910 community-based older adults to investigate relationships between cognitive reappraisal, depressive symptoms, and social support. Depressive symptoms were associated with lower levels of perceived social support, and the ability to engage in cognitive reappraisal was associated with higher levels of perceived social support. Notably, cognitive reappraisal moderated the negative consequences of depressive symptoms on perceived social support. The authors concluded that utilization of effective ER strategies can help to address the negative consequences of depression on social support among older adults. Such findings, if replicated, demonstrate the promise of specifically targeting ER as part of a therapeutic efforts aimed at improving the clinical symptoms and associated interpersonal/functional consequences of LLD.

### Neuromodulation

In the last 15 years, there has been a notable increase in neuromodulation research, especially as it pertains to ER. This usually takes the form of interventions for clinical symptom reduction and not ER specifically, though some direct work on ER has been done. Most of the relevant neuromodulation research centers around transcranial magnetic stimulation (TMS) and transcranial direct current stimulation (tDCS), as opposed to the relatively more invasive electroconvulsive therapy (ECT), vagal nerve stimulation (VNS), and deep brain stimulation (DBS). Such targeted neurostimulation therapies aim to modulate functioning of regions in the prefrontal-subcortical pathways relevant to ER. The nature of these devices, paired with the location of relevant neuroanatomical structures, has dictated targets located in prefrontal regions of the cortex, most commonly the dlPFC [54], with a more recent push to begin targeting more medial regions of the PFC [55]. While the exact mechanisms of action in reducing symptoms of depression are unknown, some models posit that improved ER is a primary mechanism in reducing negative affect [56].

## CONCLUSIONS & FUTURE DIRECTIONS

Emotion dysregulation is a key feature of mood disorders and is a target for most forms of behavioral and neuromodulation therapies aiming to reduce depression and improve positive affect. Given known age-related differences in ER strategy deployment and limited studies of ER in LLD and OABD, the interaction of age and mood on ER is not entirely clear, although targeting improvement of EF may have a secondary effect on improving ER. To address gaps in knowledge, it is important for future studies to consider including older adults with depression and powering samples to include age stratification. Future work may also consider adapting strategies known to be effective for modulating in ER in younger adults with depression for a LLD sample, with greater cognitive heterogeneity. Such approaches might consider integrating strategies from interventions such as PATH that bolster EF-based skills in older people with depression and cognitive impairment and consider the importance of social engagement to outcomes. In addition, future research on the nature and mechanisms of ER dysfunction in OABD, as well as effective interventions targeting ER, would represent a substantial contribution to the field. Lastly, it is important to note that, although group-level data point to several interesting trends with respect to how ER changes with age on average, as outlined above, these broad patterns are not a certainty at an individual level. Myriad different factors might account for individual differences in how ER strategy use changes with age. Such factors may include, for example, variability in neurotransmitter systems, brain structure and function, cognitive abilities and reserve, baseline personality organization and function, and clinical factors that are unique to an individual’s specific LLD or OABD syndrome (e.g., illness course, treatment history, symptom profiles, etc.). Thus, it will be important for future research to not only further characterize between-group differences that reflect common or average ER patterns associated with specific diagnostic categories (e.g., LLD vs OABD vs healthy aging), but to also elucidate sources of within-group variability that might point to specific, individual-level factors that can influence ER trajectories over time.

## Data Availability

No data were generated from the study.

## ABBREVIATIONS

CCN: cognitive control network
dACC: dorsal anterior cingulate cortex
DLPFC: dorsolateral prefrontal cortex
EF: executive function
ER: emotion regulation
LLD: late life depression
OABD: older adult bipolar disorder
VLPFC: ventrolateral prefrontal cortex
VMPFC: ventromedial prefrontal cortex
